# From Target Discovery to Spatial Delivery: A Translational Roadmap for Gene-Targeted Therapies in Glioma

**DOI:** 10.3390/pharmaceutics18101299

**Published:** 2026-10-14

**Authors:** Hao Cheng, Yingchao Zhang, Jinbo Zhu, Yue Yang, Jiaqi Lin

**Affiliations:** 1 MOE Key Laboratory of Bio-Intelligent Manufacturing, School of Bioengineering, Dalian University of Technology, Dalian 116024, China; chenghao@cpu.edu.cn (H.C.); zyc1113@mail.dlut.edu.cn (Y.Z.); 2 School of Biomedical Engineering, Faculty of Medicine, Dalian University of Technology, Dalian 116024, China; jinbozhu@dlut.edu.cn; 3 Cancer Hospital of Dalian University of Technology (Liaoning Cancer Hospital and Institute, Cancer Hospital of China Medical University), Shenyang 110043, China

**Keywords:** glioma, gene-targeted therapy, drug delivery, spatial pharmacology, blood-brain barrier, pharmacodynamics, clinical translation

## Abstract

Diffuse gliomas are increasingly defined by molecular alterations, yet the clinical translation of gene-targeted therapies remains constrained by a persistent mismatch between target biology and spatial pharmacology. In this review, we connect target discovery to clinical development by integrating spatial and longitudinal tumor biology with payload selection, delivery engineering, biomarker design, manufacturing, and mechanism-driven trials. Recent clinical practices in locoregional CAR-T cells, oncolytic and gene-delivery viruses, tumor-homing myeloid cell gene therapy, mutation- and neoantigen-directed vaccination, RNA nanomedicines, and emerging genome-editing strategies suggest that vehicle and route of administration dramatically change drug exposure, therapeutic index, immune activity, and resistance. We therefore propose the concept of “delivery-adjusted targetability,” in which a candidate target is judged not only by biological dependency but also by clonality, longitudinal stability, normal-brain expression, anatomical distribution, modality compatibility, and achievable target engagement. We further argue that the clinically actionable unit in diffuse glioma is often a target-anatomical compartment pair rather than a molecular target considered in isolation. Moreover, we advocate target-payload-vehicle-route-biomarker co-design and a stage-gated evidence package. This framework provides a potential way to compare otherwise dissimilar platforms, distinguish target failure from delivery failure, and reject biologically attractive but spatially implausible programs earlier. Treating molecular dependency and spatial access as a single design problem can make precision neuro-oncology more experimentally falsifiable and clinically actionable.

## 1. Introduction

The molecular taxonomy of diffuse gliomas has changed more rapidly than their therapeutic outcomes. The fifth edition of the World Health Organization (WHO) Classification of Central Nervous System Tumors established an integrated framework in which adult-type diffuse gliomas are defined primarily as astrocytoma, IDH-mutant; oligodendroglioma, IDH-mutant and 1p/19q-codeleted; or glioblastoma, IDH-wildtype, while pediatric-type diffuse high-grade gliomas include biologically distinct entities such as diffuse midline glioma, H3 K27-altered, and diffuse hemispheric glioma, H3 G34-mutant [1]. This molecular reclassification has improved diagnostic precision and clarified the biological meaning of recurrent alterations, but the therapeutic implications have been uneven. In glioblastoma in particular, extensive genomic characterization has produced a long list of potentially actionable alterations without a corresponding transformation in durable disease control [2,3].

The translational problem is not simply that glioma lacks targets. A molecular lesion may be targetable in biochemical terms yet clinically unactionable because it is subclonal, spatially restricted, unstable under treatment pressure, inaccessible to the chosen therapeutic modality, or shared with vulnerable normal neural tissue. Diffuse glioma anatomy amplifies these constraints. Drug exposure differs between the contrast-enhancing core, the infiltrative non-enhancing margin, perivascular and white-matter niches, the postoperative cavity, and cerebrospinal fluid (CSF) spaces. The blood-brain barrier (BBB) and blood-tumor barrier (BTB) are heterogeneous rather than binary, and vascular permeability within an enhancing mass does not imply adequate exposure of infiltrating tumor cells outside that region [4]. Thus, a therapy can be molecularly precise yet pharmacologically incomplete [4,5,6].

This distinction has become increasingly important as gene-targeted therapeutics diversify. The field now encompasses RNA interference, antisense and other transcript-directed medicines, CRISPR-based genome or epigenome editing, genetically engineered immune cells, viral gene-delivery and oncolytic platforms, and genetically informed vaccination strategies [7,8,9,10]. These modalities differ profoundly in cargo size, duration of activity, reversibility, intracellular versus cell-surface target requirements, immunogenicity, manufacturing complexity, and the feasibility of repeat dosing. Delivery therefore cannot be treated as a generic final step after payload selection. For a gene medicine, the vector, route, tissue distribution, and pharmacodynamic readout are often inseparable from the mechanism of action itself [7,8].

Recent reviews have provided high-quality syntheses of targetable alterations in glioblastoma, gene-delivery platforms, and nucleic-acid therapeutics [2,7,8]. This review complements, rather than duplicates, these efforts by examining the full clinical translation pathway—from identifying and validating molecular vulnerabilities, to selecting a therapeutic payload and delivery system, to route-specific pharmacology, pharmacodynamic biomarker design, clinical testing, and resistance monitoring. We use the term gene-targeted therapy broadly for interventions whose therapeutic logic is determined by a genomic, transcriptomic, or genetically engineered mechanism; we discuss genotype-defined small-molecule therapies only as translational comparators because they illustrate how clonal target selection, CNS exposure, molecular enrollment, and pharmacodynamic validation can be aligned. A distinguishing feature of our approach is that we evaluate target selection and delivery as a single development problem. We therefore use a target-payload-vehicle-route-biomarker co-design framework and introduce two linked concepts. “Delivery-adjusted targetability” considers biological dependence together with clonality, longitudinal stability, normal-CNS safety, spatial distribution, modality compatibility, and realistically achievable exposure; the “target-anatomical compartment pair” recognizes that the same molecular target poses different therapeutic problems in the resection cavity, infiltrative margin, ventricular/CSF space, or deep midline brain. Spatial multi-omic and longitudinal studies provide the biological rationale for this integration by showing that target abundance, cell state, microenvironmental context, and treatment response vary across both space and time [5,11,12]. These concepts are intended as practical design tools rather than validated quantitative scores. Collectively, our review aims to shift translational decision-making from whether a gene is theoretically targetable to whether the clinically relevant target-bearing cells can be reached, engaged at a tolerable exposure, and monitored in patients, thereby providing a common framework for interpreting both successful translation and recurrent modes of failure.

## 2. From Molecular Alterations to Clinically Actionable Targets

### 2.1. Molecular Entities and Translational Target Classes

WHO CNS5 makes clear that “glioma” is not a single molecular disease [1]. Adult IDH-mutant gliomas, IDH-wildtype glioblastoma, and pediatric-type high-grade gliomas have distinct evolutionary histories and target landscapes. Molecular testing guidelines have accordingly shifted toward entity-specific, therapy-oriented interpretation rather than indiscriminate sequencing [3]. For translational development, a second layer of classification is useful. Targets can be grouped according to how they can be therapeutically accessed and how likely they are to persist across space and time [1,3].

Shared clonal driver alterations are theoretically attractive because they can mark a large fraction of malignant cells and may remain conserved during progression (Table 1). IDH1 R132H is the canonical example in adult diffuse glioma and can be exploited through enzyme inhibition, mutation-specific vaccination, or potentially mutation-selective genetic strategies [13,14,15]. H3 K27 alterations similarly define a biologically coherent diffuse midline glioma population and have enabled both genotype-directed systemic therapy and antigen-linked cellular approaches [16,17,18]. Yet clonal occurrence alone is insufficient. Conventional CARs cannot directly detect intracellular driver mutations, and peptide-MHC presentation depends on HLA context and antigen processing. The optimal payload therefore depends on the target’s molecular topology, not merely its prevalence [13,14,16,18].

Tumor-associated surface antigens provide a more direct address for engineered immune cells but are often less genetically stable. IL-13Rα2, EGFRvIII/EGFR, GD2, B7-H3, and EphA2 illustrate the spectrum from highly tumor-associated to more broadly expressed antigens (Table 1). Early clinical experience shows these antigens can support locoregional CAR-T activity. Still, such clinical experience has also exposed antigen escape, heterogeneous expression, and on-target/off-tumor risk as central design constraints [18,19,20,21,22,23,24]. A surface antigen that is present in 80% of a diagnostic specimen may be a worse therapeutic address than a less abundant target if the latter is spatially concentrated in the compartment responsible for recurrence or can be paired with a bystander mechanism that extends killing beyond antigen-positive cells [25,26].

A third class comprises transcriptional, epigenetic, and cell-state dependencies (Table 1). Such targets can be biologically compelling because they reflect core regulatory programs rather than single receptor abundance. BRD8-mediated epigenetic control of the p53 network is one example of a non-mutational dependency revealed by functional genomics [27]. In principle, RNA therapeutics, CRISPR interference, epigenome editing, or vectorized gene-regulatory constructs can reach this class of intracellular liabilities. The challenge is that transcriptional states are plastic, may be shared with normal neural or immune cells, and often require high intracellular delivery efficiency to produce a meaningful population-level effect [10,27,28].

The microenvironment itself constitutes a fourth target class (Table 1). Glioblastoma is characterized by abundant myeloid cells, lymphocyte dysfunction, hypoxia, extracellular-matrix remodeling, and treatment-induced immune suppression [29,30]. Recent single-cell and spatial studies have moved beyond the oversimplified M1/M2 macrophage model and identified multiple context-dependent myeloid programs with distinct ontogenies and immunomodulatory functions [29]. This matters therapeutically because a gene-targeted intervention can be designed either to kill malignant cells directly or to reprogram the ecosystem that supports them. Tumor-homing myeloid progeny engineered to secrete IFN-α represent an emerging clinical example of the latter strategy [31].

Finally, cancer neuroscience has expanded the target landscape beyond the tumor cell and immune niche (Table 1). Glioma cells form electrically and chemically integrated networks with neurons and with one another; glutamatergic synapses and activity-dependent signaling can promote tumor growth and invasion [32,33]. More recent work implicates C1QL1-BAI3-RAC1 signaling in tumor-microtube expansion and neural synaptic remodeling [34]. At the same time, a 2026 study identified a therapeutically exploitable BDNF-TrkB/NTRK2 axis at the tumor-brain interface and coupled that target directly to postoperative intracavitary siRNA delivery [35]. This is particularly instructive: the target was not developed independently of its anatomical context. Instead, the invasive interface defined both the biological vulnerability and the appropriate delivery route [35,36].

### 2.2. Spatial and Longitudinal Instability of Therapeutic Targets

A single diagnostic biopsy is an incomplete map of a diffuse glioma. Single-cell analyses have shown that glioblastoma cells occupy recurrent neural-progenitor-like, oligodendrocyte-progenitor-like, astrocyte-like, and mesenchymal-like states, with genetic alterations biasing, but not fixing state occupancy [37]. Spatial profiling subsequently demonstrated that these programs are layered across distinct anatomic and microenvironmental regions rather than randomly mixed [38]. This architecture implies that target abundance measured in one sample can reflect sampling location as much as a stable tumor-wide property [5].

Longitudinal studies add a second dimension. Integrative single-cell profiling of matched primary and recurrent glioblastomas shows that recurrent ecosystems do not follow a single stereotyped trajectory; malignant states, immune composition, and treatment-associated programs can diverge substantially between patients [39]. Extrachromosomal DNA (ecDNA) creates an additional source of rapid oncogene adaptation. Spatial analyses of untreated glioblastoma have demonstrated heterogeneous amplification patterns involving EGFR, PDGFRA, and other oncogenic drivers, with ecDNA providing a substrate for dynamic copy-number selection [40]. For therapies directed at amplified oncogenes, therefore, apparent high-level expression at diagnosis does not guarantee stable target retention after selective pressure [12,41].

The microenvironment is similarly dynamic. Local cues shape myeloid-cell states, including hypoxia, cytokines, tumor genotype, and dexamethasone exposure [29]. Large-scale spatial and single-cell analysis in 2026 further resolved recurring malignant cellular communities and their relationships with neural and immune compartments [42]. Taken together, these studies argue against treating heterogeneity as a descriptive inconvenience. Heterogeneity is an active mechanism of therapeutic failure: it governs which cells are exposed, which are target-positive, which can enter alternative states, and which survive to seed recurrence [11,12].

For clinical development, this means that target validation should be longitudinal and multiregional whenever feasible. A translationally persuasive target package should ask four questions. Is the target present in the cells that must be eliminated? Is it present across the regions that drive recurrence? Does it persist after standard treatment and after direct selective pressure? And is its expression sufficiently distinct from vulnerable normal CNS cells to permit the intended modality and route? The answers can differ even for targets with similar bulk-tumor prevalence [43].

### 2.3. A Delivery-Adjusted Targetability Framework

**Figure 1:**
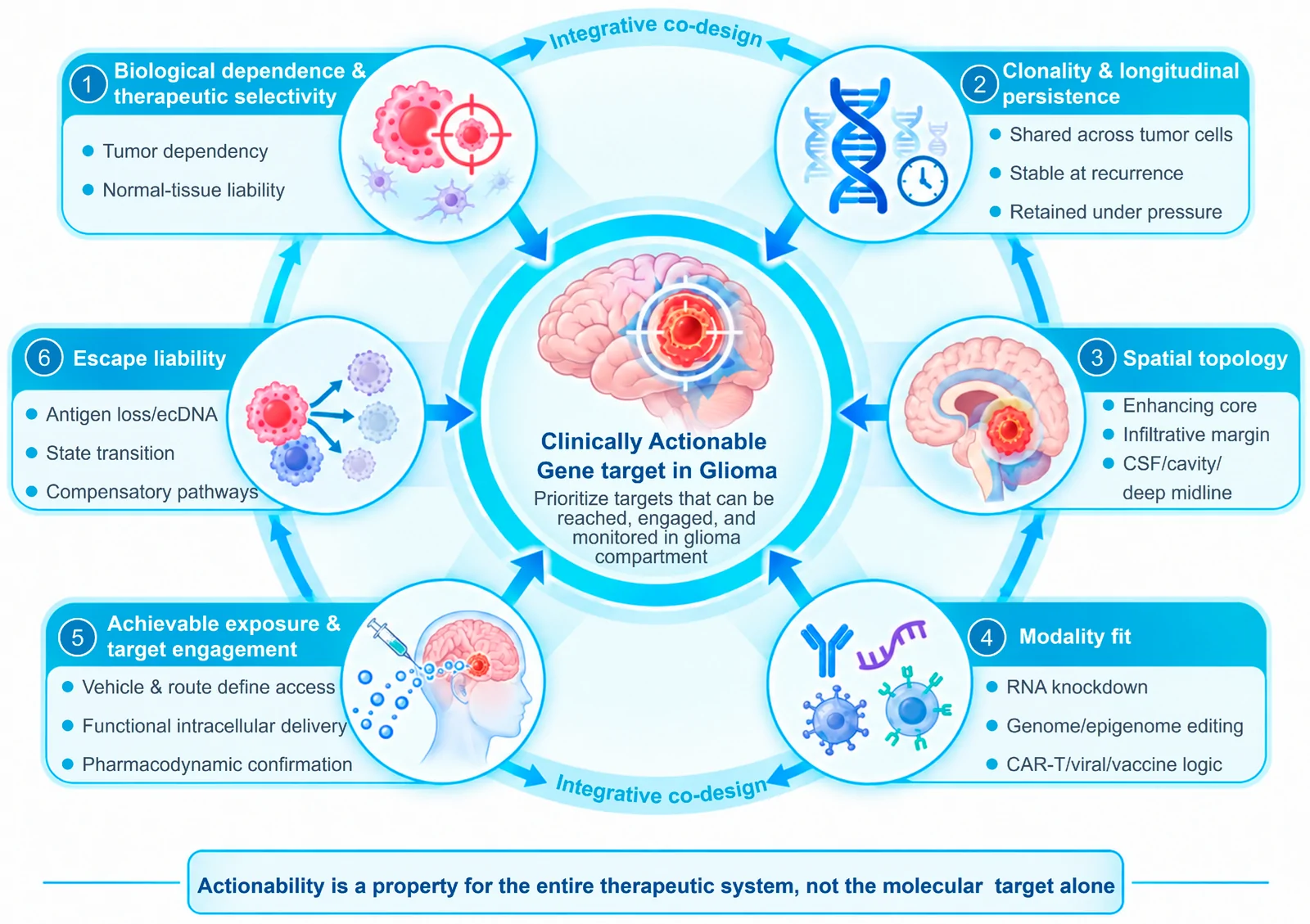
Delivery-adjusted targetability: a six-dimensional framework for clinical actionability evaluation.

We propose delivery-adjusted targetability as a conditional, six-domain assessment applied to a defined clinical setting and target-anatomical compartment pair. (1) Biological dependence and therapeutic selectivity ask whether perturbing the target produces a meaningful tumor effect without unacceptable normal-tissue injury. (2) Clonality and longitudinal persistence establish how many malignant cells carry or express the target and whether it survives standard treatment and direct selective pressure. (3) Spatial topology identifies where target-positive cells reside relative to the enhancing core, infiltrative margin, CSF, deep structures, and surgically accessible spaces. (4) Modality fit determines whether the required intervention is intracellular knockdown or editing, cell-surface recognition, transgene expression, or paracrine immune modulation. (5) Achievable exposure and target engagement ask whether the selected vehicle and route can deliver a functional payload to the relevant cells at a tolerable dose. (6) Escape liability evaluates antigen loss, pathway redundancy, cell-state switching, and ecDNA-mediated adaptation [5,6,12]. The first two domains establish biological priority; the next three test whether the product can act in the disease-defining compartment; the last anticipates how efficacy may be lost (Figure 1).

The framework is not intended as a universal numerical score. It is a stage-gated evidence map that exposes which assumption limits a program and what experiment should follow. An intracellular dependency concentrated at the postoperative margin may favor transient local RNA delivery over systemic editing; a surface antigen distributed along CSF-contacting disease may favor repeated locoregional cellular therapy; and a heterogeneous target may require an engager, oncolytic bystander effect, or logic-gated multi-antigen circuit. The biologically strongest target can therefore be the weaker development candidate when the compartment that drives progression cannot be covered. Conversely, an anatomically reachable target with moderate prevalence may become more valuable when the product provides amplification, migration, or bystander activity. Target quality is conditional on the exposure field that can be created and verified in patients.

## 3. Gene-Targeted Therapeutic Modalities: Matching Payload to Disease Biology

### 3.1. Transcript Modulation and RNA Therapeutics

**Figure 2:**
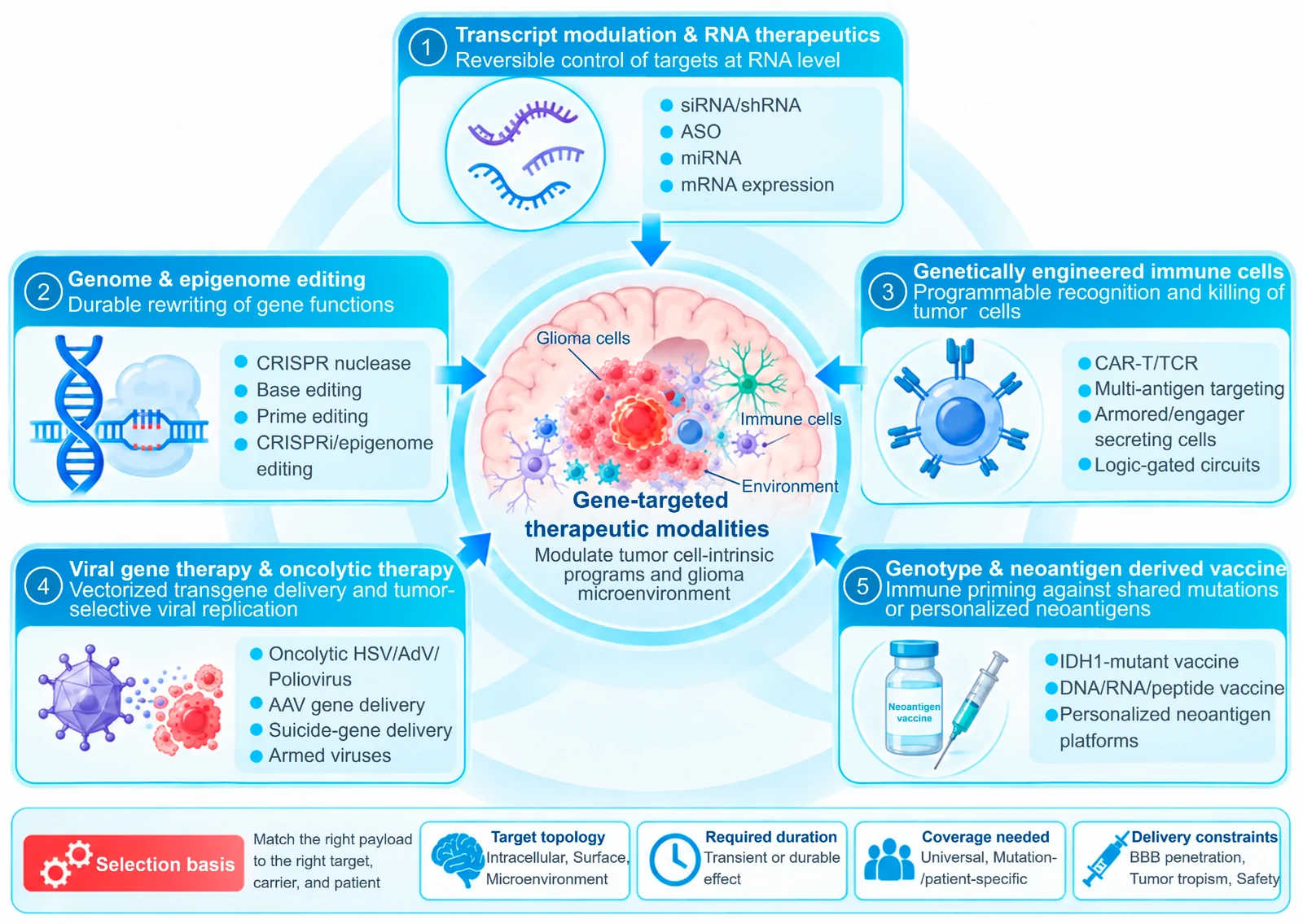
Major gene-targeted therapeutic modalities in diffuse glioma.

RNA therapeutics are attractive in glioma because they can modulate intracellular targets without permanently altering the genome. The therapeutic spectrum includes siRNA and shRNA for transcript knockdown, antisense oligonucleotides for splice or transcript control, miRNA replacement or inhibition, and mRNA for transient protein expression (Figure 2). Their pharmacology is tunable: activity can be dose-dependent and reversible, and multiple RNAs can in principle be combined. These properties suit targets for which permanent editing would create unacceptable safety risk or where repeated adjustment may be desirable as the tumor evolves [8,9].

The principal obstacle is not sequence design but productive cytosolic delivery. A nucleic-acid particle may enter a tumor cell without releasing enough intact cargo from the endolysosomal system to produce pharmacologic activity. Systemic administration adds further barriers including serum interactions, reticuloendothelial clearance, hepatic accumulation, vascular extravasation, and heterogeneous BBB/BTB permeability. These constraints make exposure-response relationships especially important in brain tumors, where plasma pharmacokinetics can be a poor surrogate for tumor-cell pharmacology [9,44,45].

The first-in-human phase-0 study of the spherical nucleic-acid construct NU-0129 provides an instructive development model. Intravenously administered gold nanoparticles carrying siRNA against BCL2L12 were detected in recurrent glioblastoma tissue and were associated with molecular evidence of target engagement [46]. The study was not designed to establish clinical efficacy; its importance lies in showing how resection-window studies can answer a more fundamental question before large efficacy trials: did the proposed delivery system actually reach human tumor tissue and perturb the intended pathway? This principle should apply more broadly to nucleic-acid therapeutics [43].

Lipid nanoparticles (LNPs) and related lipid-based systems remain among the most adaptable nonviral vehicles for RNA medicines because ionizable lipids can protect RNA cargo, facilitate cellular uptake, and support endosomal escape [9,44]. Importantly, recent glioblastoma studies increasingly test these systems with RNA payloads rather than treating the particle as a generic carrier. Polymer-locking fusogenic liposomes have delivered MDK-directed siRNA across the BBB and restored temozolomide sensitivity in orthotopic models; other studies have used BBB/BTB-penetrating siRNA nanocarriers to reshape immunosuppressive signaling, cancer-cell-membrane-coated nanoparticles to deliver STAT3 siRNA in drug-resistant glioblastoma, and peptide-conjugated LNPs to improve PLK1-siRNA delivery [47,48,49,50]. Virus-mimicking nanoparticles likewise illustrate how protein conformation and lipid nanoenvironments can be co-engineered to achieve cell-selective siRNA delivery [51]. More broadly, BBB-crossing lipid chemistries capable of systemic mRNA delivery to the CNS support the feasibility of extending RNA formulations beyond local administration. However, tumor-selective uptake and productive cytosolic release remain separate requirements [52]. These studies sharpen the translational question for RNA therapeutics: not whether RNA can be packaged, but whether sufficient intact RNA reaches the relevant glioma cell population and produces a measurable, repeatable pharmacodynamic effect.

### 3.2. Genome and Epigenome Editing

CRISPR-Cas nuclease editing uses a guide RNA to recruit a Cas nuclease to sequence-matched genomic DNA adjacent to an appropriate protospacer-adjacent motif (Figure 2). Double-strand cleavage is then repaired predominantly by non-homologous end joining, producing insertions or deletions that can disrupt a coding sequence; homology-directed repair can install defined sequence changes when a donor template and a permissive repair state are available, but it is inefficient in many clinically relevant settings. For cancer therapy, nuclease editing is therefore most naturally suited to loss-of-function interventions, whereas uniform allele correction across a diffuse tumor is substantially more demanding [10,53].

Base and prime editors reduce reliance on double-strand breaks and therefore expand the range of sequence-level interventions that may be considered for glioma [10,54,55,56,57]. Cytosine base editors couple a catalytically impaired or nickase Cas protein to a cytidine deaminase to install defined C-to-T transitions within a narrow editing window. In contrast, adenine base editors use an evolved adenine deaminase to mediate A-to-G conversion. Prime editors combine a Cas9 nickase with reverse transcriptase and a prime-editing guide RNA that both recognize the target and encode the desired sequence, permitting a broader spectrum of substitutions and small insertions or deletions without a donor DNA template. These platforms avoid a programmed double-strand break but do not eliminate risk: editing-window and PAM constraints, bystander or off-target changes, editor size, delivery stoichiometry, and heterogeneous editing efficiency remain important determinants of translational feasibility. A glioma-specific example is adenine base editing of IDH1 R132H in patient-derived models, organoids, and in vivo systems using a dual-AAV split-intein strategy. Editing reduced mutant IDH1 protein and 2-hydroxyglutarate, but highly edited cells showed increased proliferation [54]. This result is mechanistically instructive: precise correction of an initiating mutation is not automatically equivalent to therapeutic benefit, and editing programs must validate the phenotypic consequence of the intended molecular correction rather than assume it.

CRISPR interference (CRISPRi) and epigenome editing provide a third logic in which the underlying DNA sequence is left intact. Nuclease-dead Cas proteins can be fused to transcriptional repressors or activators, or to enzymes that write or erase DNA and histone marks at selected regulatory elements [10,28]. This approach is particularly attractive for dosage-sensitive, transcriptional, or drug-resistance programs in which permanent sequence alteration is unnecessary. A 2025 glioblastoma study provides a useful proof of principle: transient CRISPRoff mRNA plus sgRNAs produced durable epigenetic silencing of MGMT and restored sensitivity to temozolomide in established and patient-derived models, including orthotopic tumors; CRISPRi screening further identified BRIP1 and FANCE as vulnerabilities that could be multiplexed with CRISPRoff to sensitize tumors to lomustine [58]. The study also illustrates a broader design opportunity for glioma: a transiently delivered editor can, in principle, create a longer-lived pharmacodynamic state. The corresponding challenge is that durable epigenetic memory, imperfect cell coverage, and glioma cell-state plasticity may produce exposure-response relationships that differ fundamentally from those of conventional reversible RNA drugs.

Direct tumor editing in glioma remains a technically compelling but predominantly preclinical strategy. Targeted LNPs have delivered CRISPR-Cas9 components in orthotopic glioblastoma models, BBB-penetrating single Cas9/sgRNA nanocapsules have achieved intratumoral PLK1 editing, and polymer-locking fusogenic liposomes have supported both MDK-directed RNA interference and CRISPR-Cas cargo delivery [47,59,60]. These studies demonstrate that editing chemistry can be coupled to brain-tumor delivery, but they do not establish whole-tumor coverage in patients. For a diffuse malignancy, editing a high fraction of cells in an accessible tumor sector does not eliminate the clonogenic and infiltrative compartments that drive recurrence.

The irreversibility of permanent editing also raises the translational threshold. Off-target sequence changes, large deletions or rearrangements, chromosomal damage including chromothripsis after double-strand breaks, immunogenicity of editor proteins, inadvertent modification of normal neural or immune cells, and heterogeneous editing across tumor sectors all require explicit assessment [61,62,63]. Consequently, the most realistic near-term applications in glioma may be those in which editor exposure can be spatially confined or made transient, or in which editing is performed ex vivo to engineer therapeutic cells rather than in vivo to modify every malignant cell. Direct in vivo editing of human glioma cells therefore remains preclinical as of September 2026, and technological feasibility in orthotopic models should not be conflated with clinical readiness.

### 3.3. Genetically Engineered Immune Cells: From Single Antigens to Programmable Recognition

Engineered immune cells solve several delivery problems by acting as living, motile, and potentially self-amplifying therapeutics (Figure 2). CAR-T cells are especially attractive for surface antigens because recognition does not require HLA presentation. Yet the glioma setting exposes limitations that are less prominent in hematologic malignancies: heterogeneous antigen density, restricted trafficking, an immunosuppressive myeloid environment, T-cell exhaustion, steroid exposure, and the risk that inflammation within a fixed intracranial volume will produce clinically significant edema or neurologic deterioration [18,19,20,21,22,23,26,30,64].

Recent platform evolution therefore focuses less on building a stronger single CAR and more on engineering information processing. Bivalent and multi-antigen constructs can reduce escape. Secreted T-cell engagers can recruit bystander T cells or redirect CAR-T activity toward antigen-heterogeneous tumor cells. “Armored” cells can locally release cytokines or modify inhibitory pathways. Synthetic Notch (synNotch) circuits add conditional logic: recognition of a priming antigen induces a secondary CAR, allowing the cell to integrate two imperfect signals and restrict potent killing to a tissue or tumor context [24]. Such circuits are conceptually important because they convert target heterogeneity from a simple exclusion criterion into an engineering problem [26].

This programmability also changes how we conceptualize delivery. For a conventional drug, the carrier’s role largely ends once the payload reaches the target tissue. For a cellular therapy, distribution includes trafficking, persistence, activation, proliferation, and cytokine secretion. Intracerebroventricular or intratumoral administration can bypass systemic trafficking barriers, but local access does not guarantee uniform parenchymal migration. Consequently, cellular pharmacokinetics should include CSF persistence, intratumoral localization, expansion kinetics, antigen-dependent activation, and the spatial pattern of cytokine release rather than blood counts alone [19,21,26].

### 3.4. Viral Gene Therapy and Oncolytic Immunotherapy

Viral platforms combine delivery with biological activity in ways that distinguish them from inert carriers (Figure 2). Oncolytic viruses can selectively replicate in tumor cells, cause direct lysis, release tumor antigens and danger signals, and reshape local immunity. Non-replicating vectors such as adeno-associated virus (AAV) can support therapeutic transgene expression with a different safety and manufacturing profile. For both classes, capsid or envelope tropism, transgene payload, transcriptional control, pre-existing immunity, repeat dosing, and route of administration determine the actual therapeutic product [65,66].

The glioma field has accumulated clinically meaningful experience with herpes simplex virus (HSV), adenovirus, poliovirus-based constructs, and replication-competent retroviral systems, including the PVSRIPO/lerapolturev poliovirus platform [65,67]. The key lesson is that viral taxonomy alone does not predict success. G47Δ, CAN-3110, DNX-2401, and pediatric G207 each use distinct combinations of replication control and immunogenicity [68,69,70,71]. Conversely, the phase III failure of vocimagene amiretrorepvec plus flucytosine illustrates that compelling early-phase biology and a rational prodrug system do not ensure an overall-survival benefit when tested against contemporary control therapy [72].

A promising direction is increasingly precise transcriptional control. Synthetic super-enhancers (SSEs) constructed from glioma stem-cell regulatory logic have been used to drive tumor-selective AAV payloads, including HSV thymidine kinase and IL-12, while limiting expression in normal brain models [73]. The 2026 SSE-7 study coupled SOX2/SOX9-responsive expression to HSV-TK/ganciclovir-mediated killing and IL-12 production. In an immunocompetent orthotopic model, tumor clearance and protection against rechallenge occurred despite reporter transduction in only approximately 20% of tumor cells, supporting an immune bystander mechanism. Selectivity was also examined in human tumor and margin slices. However, activity in fetal neural stem-cell cultures and dependence on signaling state indicate that regulatory selectivity remains conditional. Normal progenitor exposure and treatment-induced state changes require further investigation. This approach is notable because it moves specificity from the vector surface into the regulatory DNA it carries. The study also provides a general design principle: when physical targeting cannot guarantee cell-type selectivity, biological logic can be layered onto the delivery system to create a second safety gate [66].

### 3.5. Genotype- and Neoantigen-Directed Vaccination

Vaccines sit at the boundary between gene-targeted therapy and immunotherapy (Figure 2). They do not necessarily alter the patient’s genome, yet their therapeutic logic can be defined by a tumor-specific mutation or a genomically selected neoantigen set. The IDH1 R132H vaccine is a leading example of a shared driver neoantigen strategy. In a phase I study of newly diagnosed IDH1-mutant astrocytoma, the peptide vaccine was feasible and immunogenic, showing that a disease-defining mutation can serve not only as a diagnostic marker but also as an immune target [13].

Personalized vaccination extends this concept to private neoantigens. A 2026 phase I study of an adjuvant personalized multivalent DNA vaccine in MGMT-unmethylated glioblastoma demonstrated the feasibility of encoding many patient-specific neoantigens in a single construct and documented vaccine-induced immune responses [74]. As with other individualized gene medicines, however, manufacturing time is part of pharmacology: a product that cannot be designed, released, and delivered before rapid progression is functionally inaccessible to the patient who needs it.

Vaccines also reveal the importance of spatial sampling. A neoantigen selected from one sector may be absent from another, particularly in a disease shaped by subclonal evolution and ecDNA. Multiregional sequencing and prioritizing truncal or functionally constrained neoantigens may therefore matter more than maximizing the raw number of predicted epitopes. The translational objective is not the most personalized vaccine possible, but the smallest set of targets that maximizes tumor-wide and longitudinal coverage [5,41].

## 4. Spatial Pharmacology and Delivery of Gene Medicines

### 4.1. Glioma as a Set of Pharmacologically Distinct Compartments

The spatial anatomy of glioma determines which delivery problem to solve. Systemically administered therapeutics encounter circulation, the BBB/BTB, the extracellular matrix, interstitial pressure, and cellular uptake barriers before reaching an intracellular target. A locally injected therapy avoids several of these steps but introduces new constraints: catheter placement, reflux, limited diffusion, heterogeneous convection, tissue injury, and incomplete coverage of distant infiltrating cells. CSF delivery can access ventricular and leptomeningeal surfaces but may produce steep gradients between fluid spaces and deep parenchyma [4,6,45].

The BBB should therefore not be treated as a single closed gate. In the enhancing core of glioblastoma, abnormal angiogenesis can produce substantial vascular leakiness. Still, permeability is heterogeneous and infiltrative tumor outside the enhancing region may remain protected by relatively intact neurovascular barriers [4]. This explains why a systemic therapy can generate impressive concentrations in resected enhancing tissue and still fail to achieve adequate control of the infiltrative margin from which recurrence arises. For gene medicines, which are typically larger and more structurally complex than small molecules, this mismatch is especially consequential [4,6].

**Figure 3:**
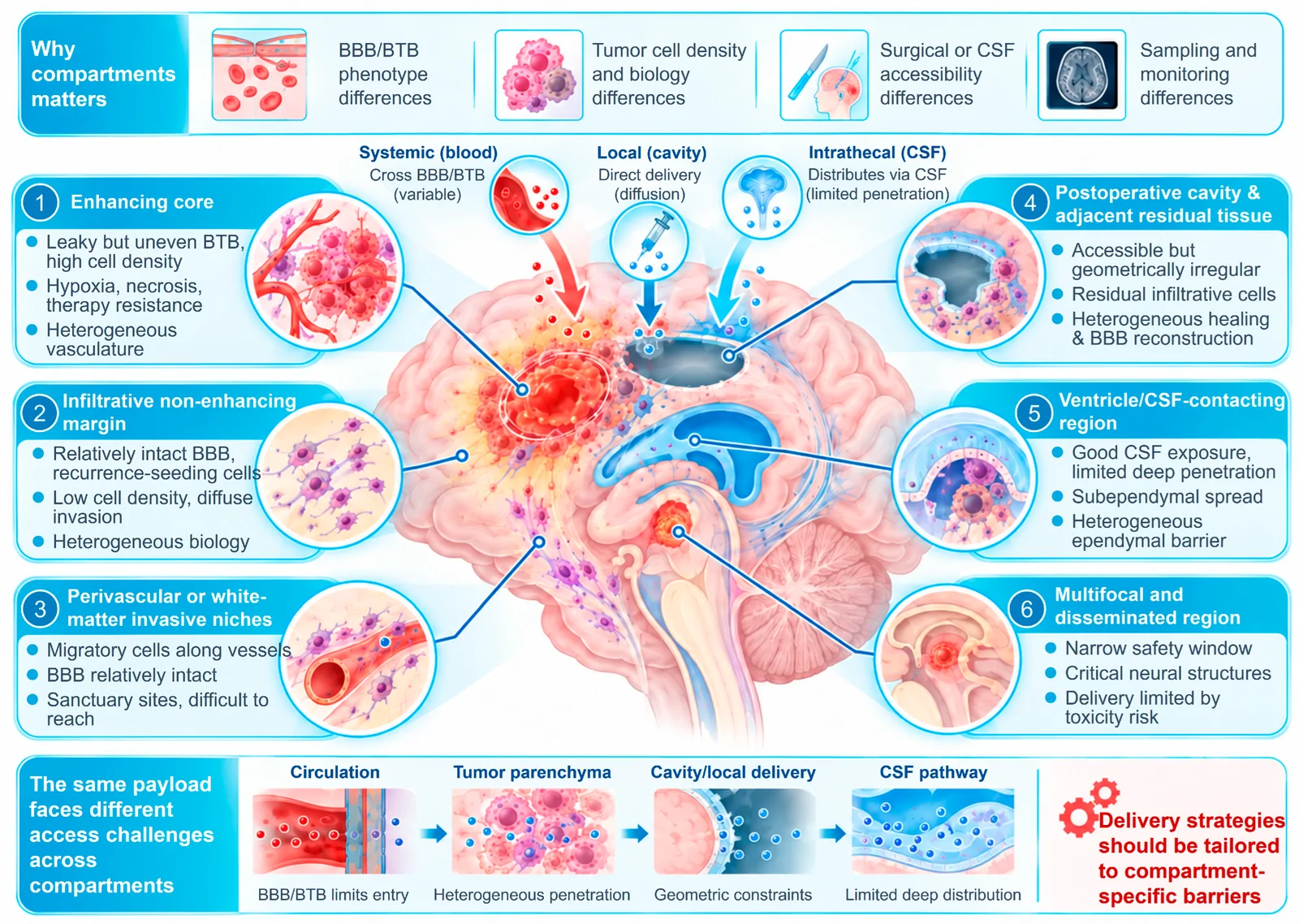
Glioma heterogeneity induces compartment-specific delivery barriers.

We propose that translational studies explicitly define the intended treatment compartment. (1) macroscopic enhancing tumor, (2) infiltrative non-enhancing margin, (3) perivascular or white-matter invasive niches, (4) postoperative cavity and adjacent residual tissue, (5) ventricle/CSF-contacting region, or (6) multifocal and disseminated region (Figure 3). This partition is intended as a pharmacological development framework rather than a fixed anatomical taxonomy. Its basis is that these regions differ along four variables that directly alter exposure and therapeutic index: vascular/BBB phenotype and interstitial transport; tumor-cell density and the biological contribution of residual or invasive cells to recurrence; physical connectivity to a surgically accessible cavity or CSF space; and the ability of a given route to achieve effective concentrations and permit sampling/monitoring of exposure in that region. Spatial multi-omic studies show that malignant states and tumor-host interactions are regionally organized. At the same time, imaging and pharmacological work demonstrate that contrast enhancement is an imperfect surrogate for barrier permeability or microscopic drug access [4,5,6,38]. Surgery further creates a transient, highly accessible but geometrically irregular residual-disease compartment, whereas CSF exposure does not imply deep parenchymal penetration. The same payload may therefore require different formulations, routes, doses, and biomarker strategies in each compartment.

### 4.2. Systemic Delivery: Broad Reach, Multiple Biological Filters

**Figure 4:**
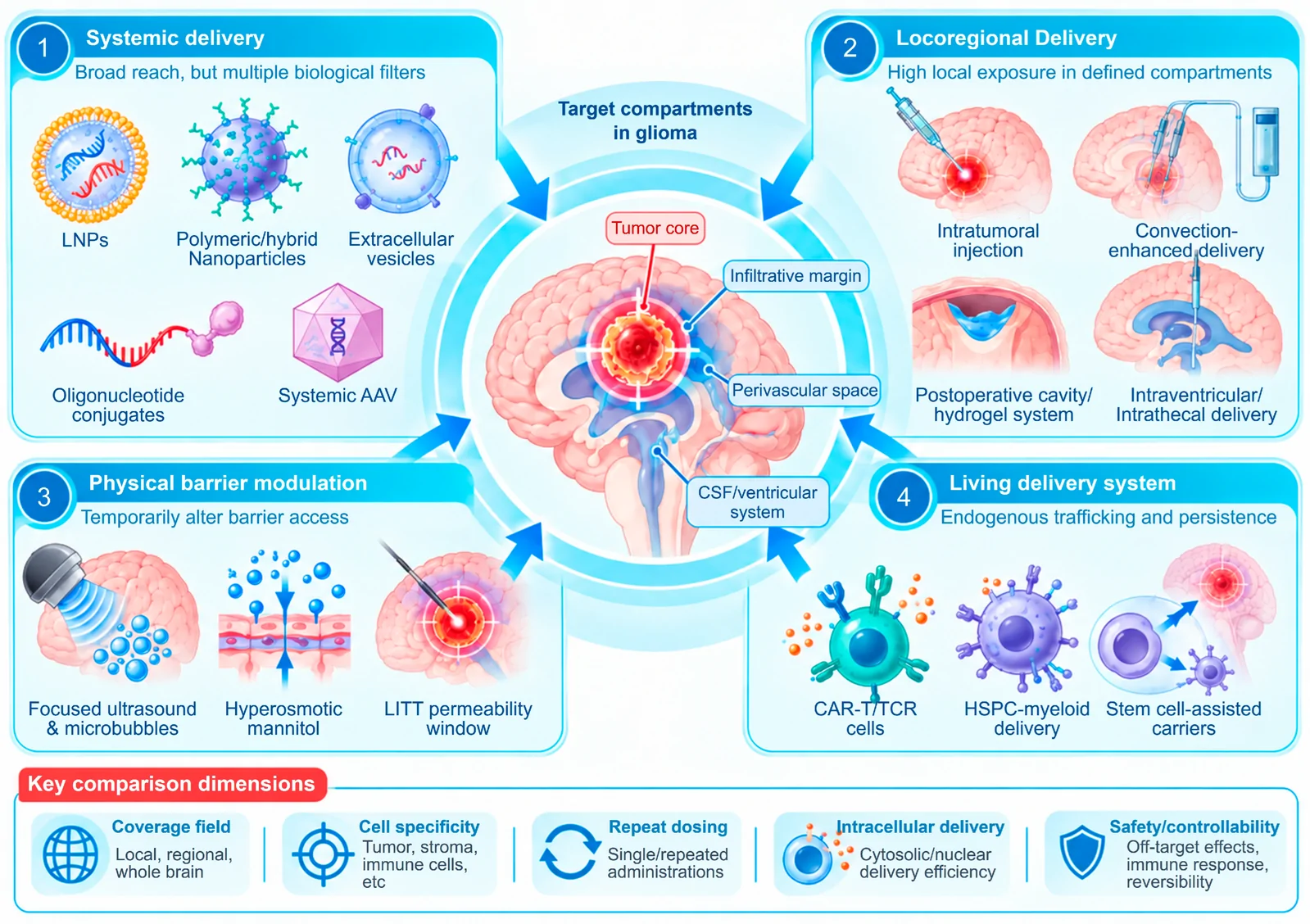
Delivery strategies for gene medicines in glioma.

Systemic delivery is attractive because diffuse glioma is not confined to one surgical target and because repeated outpatient administration is feasible. The price of broad reach is exposure to the entire organism: nanoparticles, viral vectors, proteins, and oligonucleotides can be opsonized, cleared by the reticuloendothelial system, sequestered by liver and spleen, or excluded by brain endothelium before encountering tumor cells. Current systemic strategies therefore exploit complementary mechanisms, including receptor-mediated or adsorptive transcytosis, ligand and antibody targeting, engineered nanoparticles and polymers, biomimetic coatings, extracellular vesicles, oligonucleotide conjugates, and capsid engineering [7,8,45,75,76]. The relevant comparison is not simply “viral versus nonviral,” but which platform can create the required exposure field, cell-type selectivity, intracellular release, and repeat-dose profile for a particular payload (Figure 4).

For LNPs, brain delivery requires simultaneous optimization at several levels: particle size and composition, ionizable-lipid chemistry, protein-corona behavior, BBB interaction, endosomal escape, and cell-type selectivity [44,45]. Recent BBB-crossing lipid chemistries have achieved broad CNS mRNA expression after intravenous dosing in preclinical models and human ex vivo brain tissue, while glioblastoma-directed formulations have incorporated targeting peptides or tumor-responsive fusogenic behavior to improve RNA delivery [47,50,52]. The dominant failure mode can therefore shift as the formulation improves: a system that initially fails because it never enters the brain may later become limited by endosomal trapping or off-target neuronal uptake. Development programs should quantify each barrier rather than report “brain accumulation” as a single endpoint.

Beyond LNPs, biodegradable polymeric and lipid-polymer hybrid nanoparticles can tune circulation time, cargo release, and ligand display. In contrast, biomimetic or membrane-coated systems attempt to reduce nonspecific clearance and exploit endogenous trafficking. Glioblastoma studies using cancer-cell-membrane-coated particles and virus-mimicking protein-lipid assemblies illustrate how these approaches can transport siRNA across BBB/BTB interfaces while adding tumor-cell selectivity [45,49,51,75]. Extracellular vesicles (including exosomes) offer another biologically derived route for RNA carriage and intercellular delivery, but source heterogeneity, variable cargo loading, rapid systemic redistribution, scale-up, and release testing remain major translational barriers [77].

Conjugate-based delivery provides a chemically simpler alternative to particulate carriers. Receptor- or transport-pathway-engaging ligands can be coupled directly to oligonucleotides or proteins to exploit endothelial transcytosis. A BBB-crossing conjugate system reported in 2025 used gamma-secretase-mediated transcytosis to transport an oligonucleotide after intravenous administration and produced gene silencing in mice and human ex vivo brain tissue [78]. Although this work was not a glioma therapy, it is relevant to gene medicines because it separates BBB transport from nanoparticle assembly and may support repeated systemic dosing of compact nucleic-acid cargos. The remaining challenge in glioma is to add tumor- and cell-selective uptake after crossing the endothelial barrier.

Systemic AAV presents an analogous trade-off between distribution and controllability. Engineered capsids can alter tropism: an AAV capsid reprogrammed to bind human transferrin receptor enabled brain-wide gene delivery in a humanized receptor model, and complementary interactome work identified LRP6 as a receptor associated with BBB transcytosis by engineered AAV capsids [79,80]. These are CNS-platform studies, not demonstrations of glioma selectivity. In cancer, anti-capsid immunity, systemic organ exposure, dose-dependent toxicity, limited repeat-dosing options, long expression kinetics, vector-genome design, and the proportion of full versus empty/intermediate capsids must be considered alongside tropism [66,81,82]. Tumor-restricted promoters, regulatable transgenes, and local administration can provide additional safety layers when disease geometry permits.

### 4.3. Locoregional Delivery: Turning Anatomy into a Therapeutic Advantage

Locoregional delivery is often framed as a workaround for inadequate BBB penetration. We argue it should instead be viewed as a deliberate pharmacologic strategy that can create an exposure window systemic administration cannot achieve. Intratumoral injection, convection-enhanced delivery (CED), postoperative intracavitary systems, and intraventricular or intrathecal dosing can produce high local concentrations while reducing systemic exposure. This is particularly relevant for cytokines, viral vectors, engineered cells, and genome-modifying agents with a narrow systemic therapeutic index [83].

CED uses a pressure gradient to distribute agents through extracellular spaces beyond the diffusion radius of a simple injection. Its translational success depends on catheter geometry, infusion rate, tissue anisotropy, leakage into CSF or resection cavities, and real-time confirmation of distribution. Thus, the device and procedure are components of the medicine [83,84]. A biologically active vector that is delivered to only a small fraction of the intended volume has failed pharmacologically even if its molecular mechanism is intact (Figure 4).

The postoperative cavity is another strategically important compartment. Surgery removes the dominant mass but leaves microscopic infiltrative disease in adjacent brain, creating a temporary opportunity for local therapy before tissue remodeling and recurrence. A recent NTRK2 siRNA study linked interface-enriched TrkB signaling to a hydrogel system integrating cRGD-mediated tumor targeting, glutathione-responsive siRNA release, and pH-sensitive matrix degradation [35]. In syngeneic and patient-derived postoperative models, TrkB silencing reduced excitatory synaptic input and tumor regrowth. Median survival in GL261 GBM-bearing mice increased from 32 to 67 days. Improvements in anxiety and memory without impaired motor function strengthen the therapeutic-index argument, but do not establish long-term human neural safety. In our framework, the remaining questions are functional knockdown throughout the human cavity margin, protection of normal TrkB-expressing cells, and coverage of distant infiltrative lesion. The broader lesson is that postoperative delivery should be designed around residual-disease geography, not simply around the convenience of an available cavity [35,43].

Intra-CSF administration extends the locoregional strategy beyond the postoperative margin. Intraventricular delivery through an implanted reservoir enables repeated dosing and serial CSF sampling. While lumbar intrathecal injection accesses the subarachnoid space without cranial catheter placement. Established intra-CSF pharmacology suggests ventricular delivery yields more uniform drug distribution, but CSF flow obstruction can compromise either route [85]. In glioma, these routes are particularly attractive for CSF-contacting or disseminated malignant lesion treatment, and for engineered cells capable of trafficking beyond the infusion site. Early CAR-T trials support repeated administration and measurable compartmental immune activity [19,20].

Taken together, locoregional delivery can improve the therapeutic index via bypassing vascular barriers, concentrating treatment within a defined compartment, and enabling repeat dosing and pharmacodynamic sampling when durable access is available [19,85]. Nevertheless, we cannot ignore the risks of invasive access and infection, and catheter geometry, heterogeneous convection, and CSF flow disturbances can leave infiltrative reservoirs untreated [84,85]. Reduced systemic exposure also does not ensure CNS safety. Local immune activation can cause edema, neurotoxicity, and obstructive hydrocephalus [18,20]. Therefore, delivery-route selection should follow the target-anatomical compartment pair and ensure the exposure field reaches disease-driving cells at a tolerable dose. Complementary routes or barrier modulation may be needed when those cells extend beyond the local exposure field.

### 4.4. Physical Modulation of Brain Barriers

Focused ultrasound (FUS) combined with circulating microbubbles can transiently and regionally increase BBB permeability through mechanically induced endothelial and tight-junction perturbation. Clinical studies using implantable ultrasound devices have demonstrated the feasibility of repeated BBB opening in recurrent glioblastoma with albumin-bound paclitaxel or carboplatin [86,87], and separate work has shown enhanced delivery of doxorubicin and antibodies together with measurable immune effects [88]. Although these trials were not gene-therapy studies, they establish an enabling principle that is highly relevant to gene medicines: barrier permeability can be modulated repeatedly in humans with image-defined spatial control [83,89].

Hyperosmotic BBB disruption represents a less focal but clinically established physicochemical approach. Superselective intra-arterial infusion of hyperosmolar mannitol increases plasma osmolality, draws water from brain endothelial cells, and transiently loosens paracellular barrier properties, allowing subsequently administered agents greater access to brain tissue [83,89,90]. Its strengths are mechanistic simplicity and the ability to expose a vascular territory; its limitations include catheter dependence, limited spatial selectivity, interpatient variability in the magnitude and duration of opening, and neurologic toxicity. Contemporary reviews of high-grade glioma BBB-disruption studies identify seizures among the most common procedure-associated adverse events, particularly in mannitol-based cohorts [89]. Thus, osmotic opening remains important proof that BBB permeability is an active therapeutic variable, but its precision-risk balance may be less favorable for repeated delivery of potent gene medicines than image-guided focal methods.

Thermal approaches create another localized permeability window. Laser interstitial thermal therapy (LITT) is primarily ablative. Still, mechanistic work published in 2026 showed that LITT transiently disrupts tight-junction integrity near the ablation zone for approximately 7 days and increases caveolae-mediated endothelial transcytosis for up to 21 days, peaking around day 14 [91]. This post-ablation interval could in principle be exploited for temporally synchronized systemic delivery. However, LITT is invasive, the spatial field is constrained by ablation geometry and thermal injury, and direct evidence for enhanced delivery of gene medicines remains preclinical or enabling rather than clinical.

For nucleic-acid therapeutics, preclinical studies have already combined ultrasound with LNPs to increase RNA delivery [92]. Translation should separate three variables that are often conflated: successful barrier opening, successful carrier deposition, and successful intracellular release of functional cargo. FUS, mannitol, or LITT may improve the first two without guaranteeing the third. Consequently, contrast enhancement or gadolinium leakage should not be treated as proof of functional gene delivery; future trials should pair barrier-modulation imaging with cargo-specific pharmacodynamic assays and route-specific safety monitoring (Figure 4).

### 4.5. Living Delivery Systems

Engineered cells blur the distinction between carrier and drug. CAR-T cells can migrate, expand, persist, and secrete effector molecules; hematopoietic stem and progenitor cells can generate tumor-homing myeloid progeny; stem cells can ferry oncolytic viruses toward infiltrative tumors. These systems exploit endogenous trafficking rather than attempting to reproduce it with synthetic nanoparticles [31,93].

The Temferon program provides a first-in-human example of this concept in glioblastoma. Autologous hematopoietic stem/progenitor cells are genetically modified so that differentiated myeloid progeny deliver IFN-α within the tumor microenvironment. In a 2026 phase I report in newly diagnosed MGMT-unmethylated glioblastoma, the strategy was feasible and generated evidence of local biological activity. However, the procedure carries the burdens and risks of stem-cell collection, genetic modification, conditioning, and hematopoietic recovery [31]. The translational significance extends beyond IFN-α, where an endogenous cell lineage can serve as a programmable distribution system for otherwise systemically toxic payloads (Figure 4).

Among living delivery platforms, tumor-homing myeloid vectors currently have the strongest mechanistic rationale for reaching infiltrative margins beyond a relatively intact BBB because they can extravasate and follow chemokine, hypoxia, and injury gradients [31]. CAR-T cells can provide a complementary modality through trafficking, expansion, and antigen-specific killing, but their penetration depends more on administration route, antigen density, and local immunosuppression [18,19,21,22]. Moreover, neural stem-cell carriers may also extend infiltrative margin distribution through tumor tropism [31]. Accessibility of the infiltrative margin is expected to reduce glioma recurrence risk.

### 4.6. Measure Delivery Rather than Assume It

A recurring weakness in neuro-oncology drug development is inferring delivery from dose, imaging enhancement, or peripheral pharmacokinetics. Gene medicines require a stronger evidence hierarchy. At minimum, development should distinguish: (1) physical presence of the vehicle or engineered cell in the intended compartment; (2) intracellular delivery of intact cargo; (3) expression or editing of the intended molecular target; (4) downstream pharmacodynamic pathway modulation; and (5) biological effects on malignant or immune cell states [43].

Window-of-opportunity and phase-0 studies are particularly valuable because surgical tissue can directly test this chain. CSF sampling adds a serial compartment for locoregional cellular and viral therapies, while vector-genome measurements, transgene RNA/protein, editing frequency, cytokine profiles, and single-cell state changes can provide orthogonal evidence of mechanism. The key development principle is simple: failure to demonstrate target engagement should halt or redesign a program before interpreting a negative survival trial as proof that the target itself was wrong [43,46].

## 5. Translational Lessons from Emerging Gene-Targeted Therapies in Clinical Studies

### 5.1. Locoregional CAR-T Therapy: Route Becomes Part of the Product

The most visible clinical advance in gene-engineered therapy for glioma has been the shift from proof-of-concept CAR-T administration toward repeated locoregional treatment. A completed phase I trial of IL-13Rα2-targeted CAR-T cells enrolled 65 patients with recurrent high-grade glioma and evaluated intratumoral, intraventricular, and combined routes [19]. The study demonstrated the feasibility of repeated regional administration and provided evidence that tumor and CSF immune pharmacodynamics can be monitored over time. Importantly, the heterogeneity of clinical responses reinforced that antigen expression alone is not a sufficient biomarker; cell persistence, trafficking, tumor burden, steroid exposure, and the surrounding immune ecosystem are likely to influence outcome [26].

An interim phase I study of bivalent CAR-T cells targeting EGFR and IL-13Rα2 extended the multi-antigen logic to recurrent glioblastoma [20]. According to the subsequent 2025 dose-escalation and exploration report, 18 patients were treated with intracerebroventricular EGFR-epitope-806/IL-13Rα2 CAR-T cells [94]. 8 of 13 patients with measurable disease had tumor regression, but only 1 patient had a confirmed partial response. Median progression-free survival was 1.9 months. Grade 3 neurotoxicity occurred in 10 of 18 patients, with no grade 4–5 events. These results distinguish early radiographic bioactivity from durable control and establish a need to interpret antitumor activity alongside neurologic toxicity. A dual-recognition product should reduce the probability that loss of one antigen is sufficient for escape. Yet multi-antigen design does not eliminate spatial heterogeneity, because the two targets can be co-expressed, mutually exclusive, or absent in different sectors. The practical implication is that pretreatment antigen mapping should evolve from a binary immunohistochemical eligibility test toward multiregional or spatially resolved assessment when feasible [26,95].

CARv3-TEAM-E illustrates a different engineering strategy. These cells recognize EGFRvIII through a CAR while secreting a T-cell-engaging antibody molecule directed against wild-type EGFR, thereby attempting to exploit the tumor specificity of EGFRvIII while extending killing to neighboring EGFR-positive/EGFRvIII-negative cells [21]. Early intraventricular experience in three patients produced rapid radiographic changes and demonstrated the platform’s biological potency, but the very small cohort precludes efficacy conclusions. Conceptually, however, this is an important move from “one receptor-one antigen” therapy toward locally programmable immune pharmacology [26].

Pediatric diffuse midline glioma has provided another important proving ground. GD2-directed CAR-T cells have shown antitumor activity after intravenous and intracranial administration in patients with H3 K27M-positive diffuse midline gliomas [18]. Intracerebroventricular B7-H3 CAR-T therapy has also been evaluated in children with diffuse intrinsic pontine glioma [22]. These studies highlight a distinctive CNS toxicity problem: inflammation can be therapeutically desirable yet mechanically dangerous in the brainstem or other confined structures. Tumor inflammation-associated neurotoxicity, edema, cytokine release, and hydrocephalus therefore need route-specific monitoring and management rather than transplantation of toxicity frameworks from hematologic CAR-T therapy [64].

In 2026, a phase I study extended intracranial B7-H3-targeted CAR-T treatment to adults with recurrent glioblastoma [23]. 15 patients received 72 infusions, and 13 underwent repeat treatment. Among 14 patients with measurable disease, 8 achieved disease control, including 1 sustained complete response. The median overall survival was 19.1 months from the first infusion. Disease control includes stable disease and should not be reported as an objective response rate. Although no dose-limiting toxicity was identified, 3 serious grade 3 treatment-related events occurred with elevated intracranial pressure, epilepsy, and depressed consciousness. CSF CAR expansion with limited peripheral activity supports regional pharmacology, but does not establish uniform parenchymal coverage. The selected, B7-H3-positive cohort and uncontrolled design prevent causal attribution of survival benefit or direct ranking against other CAR-T products. The next challenge is to convert this feasibility into durable control across antigen-positive and antigen-negative disease reservoirs.

Logic-gated circuits may become one route to that goal. Preclinical synNotch systems use a tumor- or CNS-associated priming signal to induce a secondary CAR, enabling “prime-and-kill” recognition while reducing constitutive receptor signaling and off-tumor activity [24]. Such designs are now moving toward clinical testing. Their broader importance is conceptual: specificity can arise from the relationship between two antigens and their spatial context, not only from the uniqueness of one antigen [26].

### 5.2. Viral and Vector-Based Gene Therapies: Clinical Activity, Immunogenicity, and the Value of Negative Trials

Oncolytic virotherapy has produced some of the most mature clinical data in glioma. In Japan, the phase II study of intratumoral G47Δ in residual or recurrent glioblastoma reported a high one-year survival proportion in a small, single-arm cohort and supported conditional, time-limited regulatory approval of the product [68]. The result established an important precedent but should be interpreted in light of study size, patient selection, and the absence of a randomized comparator. For the field, its strongest lesson may be operational: repeated intratumoral viral administration can be incorporated into neuro-oncology practice [65].

CAN-3110 provided a complementary mechanistic lesson. In a first-in-human study of recurrent glioblastoma, treatment linked viral replication and oncolytic immunoactivation with immune remodeling and clinical outcome, with pretreatment HSV serologic status emerging as a potentially relevant factor [69]. This illustrates why antiviral immunity should not be viewed only as a delivery obstacle. Depending on platform and timing, pre-existing or induced immunity can alter intratumoral inflammation, viral persistence, and the balance between direct oncolysis and immune-mediated tumor control [65].

The DNX-2401 plus pembrolizumab phase I/II study further emphasized this dual mechanism [70]. The combination was feasible and associated with durable responses in a subset of patients, but it did not meet the prespecified response-rate efficacy benchmark. That nuance matters. Early-phase glioma trials frequently generate striking individual responses, yet population-level efficacy may remain modest. A translational review should therefore separate evidence of biological activity from evidence that a regimen improves clinically meaningful outcomes.

Oncolytic HSV-1 G207 has also been evaluated in pediatric high-grade glioma, showing that direct viral administration is feasible in children and can induce local immune activation [71]. Neural stem-cell-mediated delivery of an oncolytic adenovirus in newly diagnosed malignant glioma demonstrated another route to improve intratumoral distribution by exploiting cellular tumor tropism [93]. These studies collectively suggest that “virus versus nanoparticle” is too coarse a comparison: a stem-cell-delivered virus, a tumor-selective replicating HSV, and an intratumoral AAV are pharmacologically distinct products even if all carry genetic payloads [67].

The randomized TOCA 5 trial is equally important because it was negative. Vocimagene amiretrorepvec, a replicating retroviral vector encoding a prodrug-activating enzyme, combined with flucytosine did not improve overall survival compared with standard-of-care treatment in recurrent high-grade glioma [72]. This result cautions against inferring late-stage efficacy from local biological activity, bystander killing, or early uncontrolled survival signals. It also argues for demonstrating spatial distribution and target-product pharmacology earlier, before large trials.

AAV is now beginning to cross the preclinical-clinical boundary in glioblastoma. Synthetic super-enhancer designs have strengthened tumor-selective regulatory logic by restricting expression to glioma stem-cell states in preclinical systems [73]. In 2026, the ADePT phase I/II study of TGX-007, an intratumorally delivered AAV-based dual-payload therapy incorporating a prodrug-activating HSV thymidine kinase and IL-12, entered clinical testing [96]. No efficacy conclusions are possible at this stage. Its significance is that non-oncolytic AAV gene delivery is being tested as an integrated, dual-mechanism product in human high-grade glioma rather than solely as a preclinical vector platform [66,81].

### 5.3. Tumor-Homing Myeloid Gene Delivery

Temferon represents a fundamentally different clinical solution to the distribution problem. Rather than forcing a synthetic carrier to cross the BBB, autologous hematopoietic stem/progenitor cells are genetically engineered so their myeloid progeny home to the tumor and deliver IFN-α locally [31]. In the 2026 interim analysis, 24 newly diagnosed patients with MGMT-unmethylated glioblastoma were treated across 8 dose/conditioning cohorts, with no dose-limiting toxicity. Durable detection of engineered cells and low circulating IFN-α support sustained delivery with limited systemic cytokine exposure. However, transplantation-associated cytopenias and infection indicate that transgene tolerability and overall procedural burden should be assessed separately. Median overall survival was 16.7 months from diagnosis, but the uncontrolled design and varied conditioning regimens limit attribution of benefit or a dose-response relationship. For this platform, engraftment is a prerequisite for distribution, but cannot ensure adequate intratumoral exposure. Quantitative evidence at infiltrative sites is needed to connect lineage persistence to effective local immune reprogramming.

This platform changes the usual definition of drug delivery. The “vehicle” is a differentiating cell lineage; biodistribution depends on hematopoietic engraftment and myeloid trafficking; a genetic cassette controls release; and toxicity includes both transgene biology and the transplantation procedure. Its translational value therefore cannot be assessed with conventional nanoparticle metrics. The relevant questions are whether tumor-homing progeny reach infiltrative compartments, how stable transgene expression remains over time, whether local payload levels exceed a therapeutic threshold without systemic leakage, and whether the manufacturing and conditioning burden is justified by the clinical benefit [31].

### 5.4. Mutation- and Neoantigen-Directed Vaccines

Mutation-directed vaccination offers a different route to spatial coverage because activated T cells can in principle survey distributed disease. The IDH1 R132H vaccine established the feasibility of targeting a shared, disease-defining neoepitope in newly diagnosed IDH1-mutant glioma [13]. The central translational advantage is target clonality: because IDH mutation is an early event in the relevant disease, antigen loss may carry a higher biological cost than loss of a dispensable surface protein. The limiting step is immunology rather than physical drug distribution: antigen processing, HLA context, T-cell trafficking, and an immunosuppressive microenvironment determine whether vaccination produces effective tumor-cell killing.

Personalized multivalent DNA vaccination in MGMT-unmethylated glioblastoma extends the strategy to private neoantigens [74]. In the 2026 GNOS-PV01 trial, 9 patients were vaccinated with 17–40 neoantigens selected from multiregional tumor sampling. Neoantigen-specific peripheral responses were detected in 6 of 7 evaluable patients, and the nonresponding patient received dexamethasone. Thus, immunogenicity supports the payload rationale but does not demonstrate tumor-wide immune control. The operational results are especially informative. Median time from radiotherapy completion to vaccination was 10 weeks, exceeding the prespecified 4-week feasibility window, and 3 patients progressed before their first dose. Median overall survival was 16.3 months from surgery, and this small, uncontrolled cohort cannot establish vaccine benefit. These findings link spatial antigen selection to two further requirements of preserving tumor antigen presentation and delivering the individualized product before progression. Turnaround time should therefore be treated as a pharmacological parameter, alongside immune competence and antigen coverage.

### 5.5. Direct RNA and Genome-Editing Therapies: A Persistent Translational Gap

Direct nucleic-acid therapy in glioma has a striking asymmetry: sophisticated preclinical delivery systems coexist with relatively limited clinical proof of intracellular target engagement. NU-0129 remains an important phase-0 precedent because it directly interrogated human tumor delivery [46]. Since then, advances in LNP chemistry, CRISPR cargo design, ultrasound-assisted BBB opening, and intracavitary RNA systems have expanded the preclinical toolkit [35,59,92]. Yet direct in vivo genome editing of malignant glioma cells has not reached the clinical maturity of engineered-cell or oncolytic-virus therapies [9,47,60].

This gap is not a failure of editing chemistry; it reflects the multiplication of probabilities required for success. A systemically administered editing nanoparticle must survive circulation, reach the appropriate brain region, cross vascular and extracellular barriers, enter the right tumor cell, escape the endosome, release an active editor, modify the target allele, and do so in a sufficient fraction of clonogenic cells without injuring normal CNS tissue. Improvements at one step may not meaningfully change the product of all steps. Local delivery and temporary editors may therefore offer a more realistic early clinical path than attempting whole-tumor systemic editing from the outset [62,63].

### 5.6. What Gene Medicines Can Learn from Genotype-Defined Drug Successes

Two recent pharmacological successes provide useful external comparators. In the INDIGO phase III trial, the brain-penetrant mutant IDH1/2 inhibitor vorasidenib prolonged progression-free survival. It delayed the next intervention in patients with residual or recurrent grade 2 IDH-mutant glioma who had not received prior radiation or chemotherapy [14]. The FDA approved vorasidenib in 2024 for eligible adults and adolescents with grade 2 IDH-mutant astrocytoma or oligodendroglioma after surgery [15]. The therapeutic lesson is not that small molecules are inherently superior to gene therapy. It is that a clonal, disease-defining alteration was paired with a brain-penetrant agent, prospective molecular selection, and a clinical setting in which the biology could be altered before highly resistant disease emerged.

Dordaviprone provides a second example. Clinical data in recurrent H3 K27M-mutant diffuse midline glioma supported accelerated FDA approval in 2025 for progressive H3 K27M-mutant disease after prior therapy [16,17]. Again, the relevant principle is alignment: molecular identity, disease compartment, drug exposure, and patient selection were tightly coupled. Gene medicines should adopt the same discipline. A technologically impressive platform cannot compensate for a weak target or an unmeasured delivery failure (Table 2).

## 6. Why Translation Fails and How the Development Path Should Be Redesigned

### 6.1. A Failure-Mode Analysis

Gene-targeted therapies in glioma can fail for fundamentally different reasons, and those reasons should not be collapsed into the generic conclusion that a target or platform “does not work.” A therapy may fail biologically because the target is subclonal, dispensable, or rapidly downregulated. It may fail evolutionarily because treatment selects an antigen-negative clone or a compensatory cell state. It may fail spatially because it achieves adequate exposure in the enhancing mass but not at the infiltrative margin. It may fail pharmacologically because the vector reaches the tissue but not the cytosol or nucleus. It may fail immunologically because myeloid suppression, lymphopenia, exhaustion, or corticosteroids blunt the intended immune mechanism. It may fail operationally because the product cannot be manufactured, released, or administered in time. Distinguishing these mechanisms is essential because each demands a different redesign [12,26,45].

**Figure 5:**
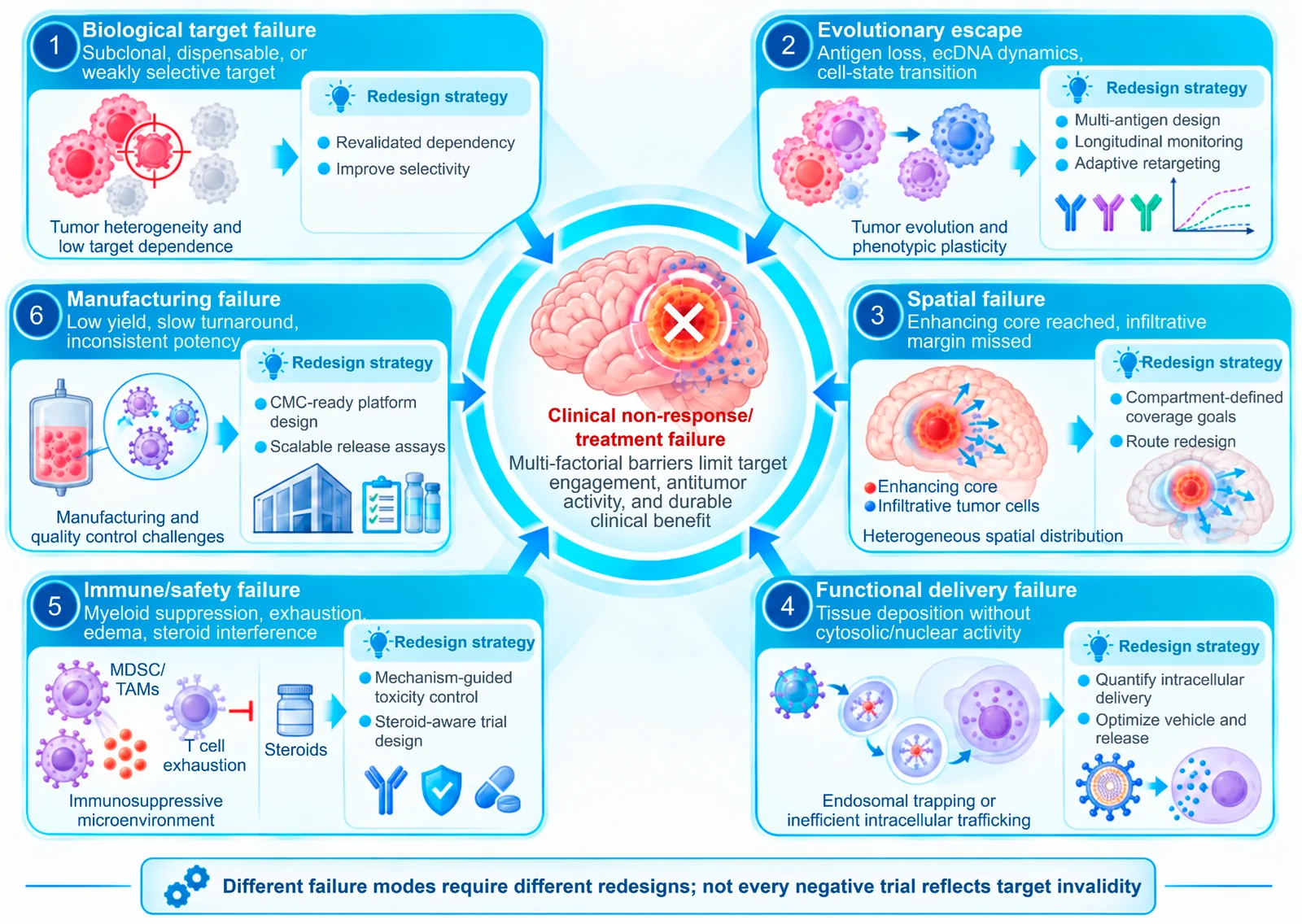
Failure modes in glioma gene therapy and matched redesign strategies.

Biological and evolutionary failure is particularly relevant for surface-antigen therapies. Antigen escape after selective pressure should be anticipated rather than treated as an unexpected complication. Multi-antigen CARs, secreted engagers, synNotch circuits, and bystander-producing viral therapies represent different solutions, but a formal estimate of escape liability should guide all. For genetically amplified targets, ecDNA adds another layer of adaptability [40]. For state-dependent targets, transitions among malignant programs can change expression without any new mutation [37,38,39]. Longitudinal biomarker sampling is therefore not merely descriptive; it is part of resistance management (Figure 5) [25,41].

Spatial failure is arguably the defining pharmacological problem of diffuse glioma. The conventional efficacy paradigm assumes that a single administered dose can be summarized by one concentration. In the brain, that assumption is often false. A therapy can achieve a high concentration in a resected enhancing lesion while leaving clinically important microscopic disease underexposed. Conversely, a CSF-delivered cell therapy can show excellent persistence in ventricular fluid but limited access to deep parenchyma. Development should therefore specify the minimum anatomical coverage required for success and measure exposure in more than one relevant compartment whenever possible (Figure 5) [4,6,45].

Immune and safety failure are tightly coupled in CNS gene therapy. Strong local inflammation may improve antigen presentation and tumor killing, yet edema in eloquent cortex, brainstem, or spinal cord can become dose limiting. CAR-T studies in diffuse midline glioma have made this tension explicit [18,22]. Viral therapies face a related trade-off: innate and adaptive antiviral responses can promote antitumor immunity but may also clear the vector and limit repeat dosing. Dexamethasone can control edema but simultaneously reshape myeloid programs and suppress lymphocyte function [29]. Steroid exposure should therefore be prospectively captured as a mechanistically relevant covariate rather than treated only as supportive care (Figure 5) [11,64].

Manufacturing failure is often underappreciated in academic development. Viral-vector yield, empty-particle content, potency, transgene stability, replication competence, cell-product phenotype, and lot-to-lot consistency determine whether an elegant laboratory system can become a medicine. Autologous CAR-T and HSPC products add leukapheresis or marrow collection, ex vivo engineering, release testing, and scheduling constraints. Personalized vaccines add sequencing, neoantigen prediction, synthesis, and quality control. These are not administrative details: they determine dose availability, treatment timing, and patient attrition (Figure 5) [81,82,97].

Finally, clinical-trial failure can obscure biological success. Imaging changes after viral or cellular immunotherapy may reflect inflammation rather than progression; conventional response assessments can misclassify early radiographic effects; and small single-arm trials are vulnerable to selection and historical-control bias. Response Assessment in Neuro-Oncology (RANO) 2.0 provides a more standardized framework for adult glioma response assessment [98]. Still, gene-therapy trials should go further by embedding mechanism-specific biomarkers and predefined rules for pseudoprogression, steroid use, and confirmatory imaging [43,98].

### 6.2. Start with a Target Product Profile, Not with a Vector

A common development pattern is platform-first approach, in which a laboratory has an AAV, LNP, hydrogel, or engineered-cell technology and searches for a glioma payload to insert into it. Platform science is necessary, but platform-first development can produce a mismatch between what the technology can deliver and what the disease requires. We recommend starting with a target product profile that specifies the molecularly defined patient population, clinical setting, anatomical compartment, intended duration of activity, acceptable toxicity, dosing frequency, and biomarker strategy before finalizing the vehicle [45,75].

**Figure 6:**
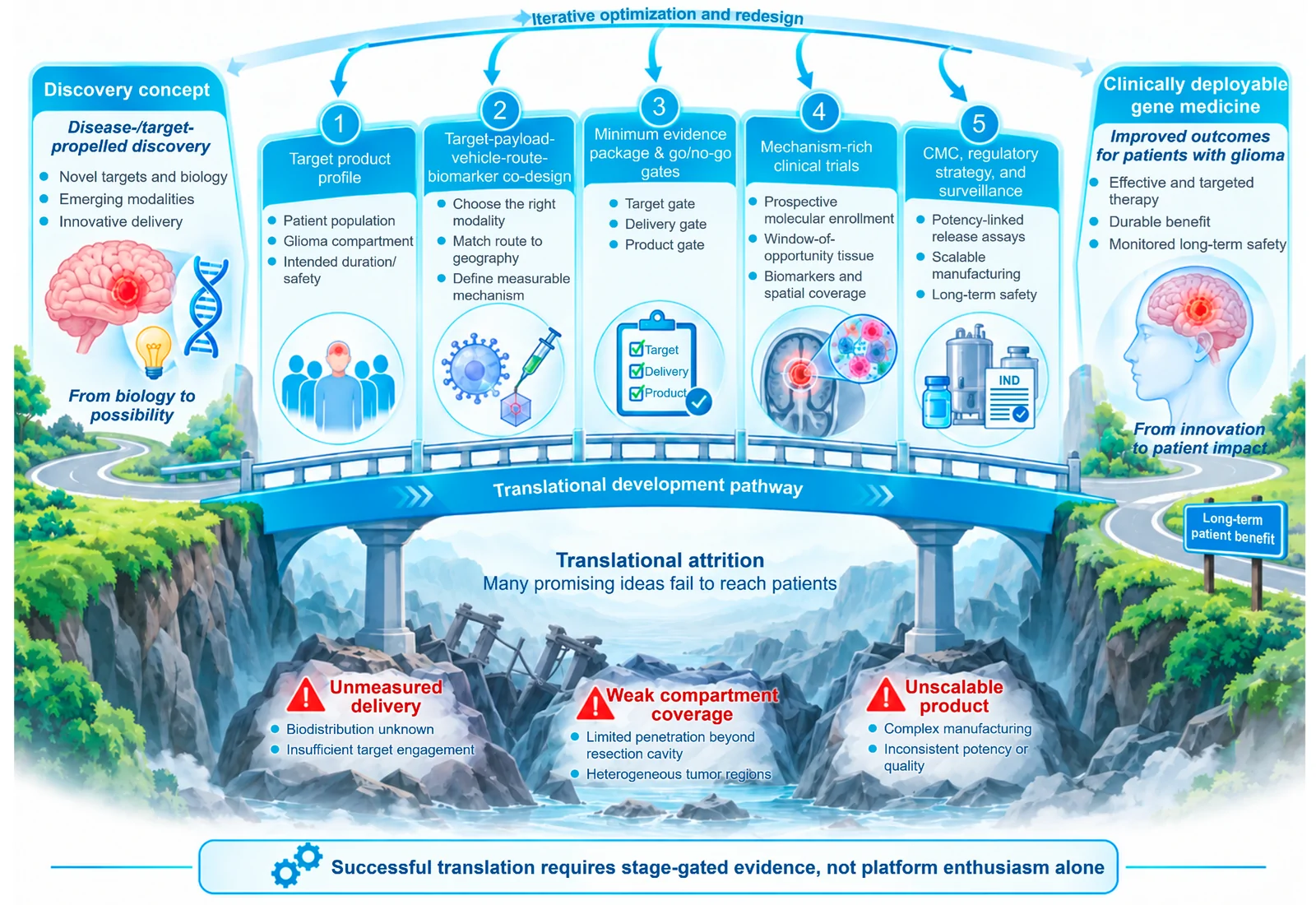
A redesigned translational development pathway for glioma gene medicines.

This sequence forces explicit trade-offs. A therapy intended for postoperative microscopic disease should be optimized for cavity-adjacent distribution and residual-cell uptake, not for maximal concentration in a bulky xenograft. A therapy for multifocal disease may need systemic or CSF access and repeat dosing even at the cost of lower peak intratumoral exposure. A therapy for a brainstem tumor may prioritize controllability and reversible activity over maximal immune potency. The route is therefore selected from disease geography, not appended after the payload is fixed (Figure 6).

### 6.3. Target-Payload-Vehicle-Route-Biomarker Co-Design

The five-part co-design framework proposed in this review can be summarized as follows. The target determines what molecular event must be changed. The payload determines whether that event requires knockdown, expression, editing, cell killing, or immune redirection. The vehicle determines which cells can receive the payload and for how long. The route determines the exposure field and much of the safety profile. The biomarker strategy determines whether the preceding assumptions can be tested in humans. A weakness in any one component can invalidate the entire chain.

Consider an intracellular dependency concentrated in infiltrative margin cells. A conventional CAR is a poor payload because the target is not surface accessible. Systemic CRISPR may be too difficult because the relevant cells sit behind a relatively intact BBB. A transient siRNA or mRNA payload delivered into the postoperative cavity may be more rational, provided diffusion and intracellular uptake can be demonstrated. Conversely, a heterogeneous surface antigen in multifocal disease might favor a living cell with multi-antigen recognition or a local engager rather than a single-antigen antibody cargo. Co-design thus converts biological description into product engineering (Figure 6).

### 6.4. A Minimum Translational Evidence Package and Go/No-Go Criteria

Preclinical evidence should be designed to falsify a product concept, not merely to produce tumor-growth curves. At minimum, the evidence package should include genetically faithful patient-derived models; immunocompetent models when the mechanism depends on immunity; human tumor organoids or ex vivo tumor/brain slices when cell-type specificity is central; explicit assessment of infiltrative-margin coverage; and, for device- or volume-dependent delivery, large-animal or anatomically relevant studies of distribution. No single model captures all aspects of human glioma, so concordance across models is more informative than maximal efficacy in one system [95,99,100].

We propose several practical go/no-go questions before first-in-human escalation: Does the product reach the clinically relevant compartment at a quantifiable exposure? Is target engagement demonstrated, not inferred? Is the normal-CNS therapeutic index acceptable at an exposure above the projected active level? Is the dominant resistance mechanism identifiable and monitorable? Can dosing be repeated if required? Can the product be manufactured reproducibly at the intended clinical scale? A “no” to any of these questions should trigger redesign rather than be deferred to an efficacy trial (Figure 6).

### 6.5. Clinical Trials Should Be Designed Around Mechanism

First-in-human gene-therapy trials in glioma should maximize information per patient. Molecular enrichment should be prospective and tied to the therapeutic mechanism. Window-of-opportunity designs should be used whenever surgery permits tissue acquisition after drug exposure. For locally delivered products, dose escalation should consider the optimal biological dose and spatial coverage, not only a maximum tolerated dose. Serial CSF sampling can be highly informative for intraventricular cellular and viral therapies, while paired tumor tissue can establish vector distribution, editing frequency, transgene expression, immune remodeling, and target suppression [43,46].

Pharmacodynamic failure should be distinguished from target failure. If an RNA therapeutic produces no target knockdown in resected tissue, a negative clinical outcome is evidence against the formulation or exposure, not necessarily the gene. If a CAR-T product expands in CSF but antigen-negative recurrence dominates, the lesson is evolutionary rather than pharmacokinetic. If a virus replicates and inflames the tumor but survival does not improve, the next question is whether immune suppression, spatial escape, or trial context prevented durable control. Mechanism-rich studies make negative results scientifically productive. Imaging should be integrated with these molecular data rather than used in isolation. Response Assessment in Neuro-Oncology (RANO) 2.0 offers a common response-assessment foundation [98], but immunotherapy and gene-therapy programs may require confirmation scans, perfusion or metabolic imaging, and predefined adjudication of inflammation-associated changes. Corticosteroid dose and timing, lymphocyte counts, anticonvulsants, and anti-edema interventions should be captured systematically because they can alter both biology and imaging interpretation (Figure 6) [6,98].

Because CAR-T, viral, and vaccine therapies can induce delayed immune-cell infiltration, edema, and contrast enhancement, clinical trials should prespecify Immunotherapy Response Assessment in Neuro-Oncology (iRANO) alongside RANO 2.0. iRANO provides a framework that requires clinical and radiographic confirmation of apparent progression during an early immune-activation window, while integrating steroid exposure and neurologic status. It should also be complemented by perfusion or metabolic imaging and, when feasible, tissue sampling. Applying iRANO does not eliminate the need to assess true progression, but it prevents premature discontinuation of active therapy and makes pseudoprogression an explicit, testable endpoint [98,101].

### 6.6. CMC, Regulatory Strategy, and Long-Term Surveillance

Chemistry, manufacturing, and controls (CMC) should enter development before, not after, efficacy optimization. Potency assays should be mechanistically linked to the intended action: a CAR-T release assay should reflect antigen-dependent function and relevant phenotype; an AAV assay should quantify both vector integrity and functional transgene expression; an RNA nanoparticle assay should measure functional delivery rather than encapsulation alone. Stability and comparability plans are particularly important for rapidly iterating academic platforms because manufacturing changes can alter biodistribution or potency [82,97].

Regulatory surveillance depends on modality. Integrating vectors and genetically modified cells may require long-term follow-up for insertional or clonal risks. Genome editors require off-target and structural-variant assessment. Replication-competent viruses require shedding and biosafety evaluation. Cytokine-expressing products require monitoring for systemic leakage and delayed inflammatory toxicity. The goal is not to eliminate all uncertainty, an impossible standard in lethal disease, but to align the surveillance plan with the irreversible or self-propagating features of the product (Figure 6) [63,66].

### 6.7. Combination Therapy Should Be Mechanism- and Sequence-Driven

Combination therapy is often invoked as a solution to glioma resistance, but empiric addition of agents can obscure mechanism and increase toxicity. Combinations should instead address a defined failure mode. Oncolytic viruses may be sequenced before checkpoint blockade to increase antigen release and local inflammation. Radiotherapy may enhance immunogenicity or alter vascular access, but timing must account for lymphocyte depletion. Local cellular therapy may control a dominant compartment while a systemic agent suppresses distant or antigen-negative disease. RNA knockdown at the resection margin may be paired with standard chemoradiation if the target mediates postoperative invasion or treatment resistance [11,70].

The sequence matters because one intervention changes the substrate for the next. Viral infection alters antigen presentation and innate immunity; corticosteroids suppress the immune context CAR-T cells require; radiation changes vascular permeability and lymphocyte abundance; targeted therapy can change surface-antigen expression. Rational combination development should therefore specify the hypothesized interaction, the optimal order, and a biomarker that confirms the first treatment created the state needed for the second.

## 7. Future Perspectives: Toward Compartment-Aware and Programmable Precision Neuro-Oncology

The next generation of glioma gene medicines will likely be defined less by a single new vector than by increasingly programmable therapeutic systems [10,24,26,73]. Multi-antigen and logic-gated immune cells, inducible cytokine expression, tumor-selective transcriptional switches, transient genome editors, and modular RNA payloads all move the field from static targeting toward conditional behavior. This is important because diffuse glioma is itself conditional: target expression, cell state, immune context, and vascular access change with location and treatment history. Emerging RNA-aggregate immunotherapy platforms further illustrate that the physical organization of an RNA therapeutic can itself contribute to innate immune activation and therapeutic function [102].

Spatial and single-cell technologies should therefore migrate from retrospective atlases into prospective therapeutic development. A clinically useful spatial assay need not resolve every cell. It should answer actionable questions: Is the target present at the infiltrative edge? Is it co-localized with the delivery receptor used by the nanoparticle? Are antigen-negative cells spatially adjacent enough for a bystander mechanism to reach them? Are the myeloid cells that suppress therapy in the same compartment that receives the vector? Such questions convert spatial biology into pharmaceutical design criteria [5,36,99].

We expect the most durable strategies to combine complementary compartments rather than pursue a single universal route. Surgery can create a postoperative window for high-concentration local RNA or viral therapy; CSF delivery can repeatedly expose ventricular surfaces and distributed CNS sites; systemic therapy can reach extracavitary or multifocal disease; and living cells can migrate beyond the initial administration site. The key is not to maximize the number of routes but to assign each route a defined biological task [83].

Adaptive treatment will also become more important. Current trials usually lock target selection before therapy and interpret recurrence as failure. A more evolution-aware model would predefine resistance biomarkers and permit retargeting or payload substitution when antigen loss, ecDNA evolution, or cell-state transition is detected. Modular CAR platforms, interchangeable RNA cargoes, and programmable vectors could make such adaptation technically feasible, but regulatory and manufacturing systems will need to evolve in parallel [12,41].

The concept of delivery-adjusted targetability offers one way to prioritize these decisions. A target should rise in priority when it is biologically important, clonally or spatially relevant, safely distinguishable from normal CNS tissue, compatible with an effective payload, and reachable through a clinically feasible route. A target should fall in priority when therapeutic exposure cannot cover the compartment that drives recurrence, even if the target is mechanistically elegant. This reverses a common order of thinking: delivery is considered during target selection, not after it.

Accordingly, the clinically actionable unit may be better defined as a target-anatomical compartment pair. “NTRK2 in the postoperative infiltrative interface,” “B7-H3 in CSF-accessible diffuse midline disease,” or “IL-13Rα2 in a locoregionally treatable recurrent cavity” is more therapeutically informative than the gene name alone. Such language forces biology and pharmacology into the same sentence, which clinical translation requires.

A final priority is manufacturability. Personalized or highly engineered products will not transform outcomes if they cannot be produced quickly, consistently, and at scale. Platform modularity should therefore be treated as a scientific objective: standardized vector backbones, reusable targeting chemistries, interchangeable payload cassettes, automated cell manufacturing, and potency assays that remain valid across product variants. In an aggressive disease, production time and access are components of therapeutic effectiveness [81,97].

## 8. Conclusions

Gene-targeted therapy for glioma is entering a phase in which the limiting question is no longer whether tumor genomes contain therapeutic opportunities. The harder question is whether those opportunities can be converted into medicines that reach the right cells, in the right anatomical compartment, at a sufficient exposure and duration, while preserving a tolerable therapeutic index. Recent clinical progress with locoregional CAR-T cells, oncolytic viruses, tumor-homing myeloid gene delivery, and mutation-directed vaccines shows this conversion is possible, but uneven outcomes across programs also show that molecular precision alone is insufficient.

We therefore propose an end-to-end translational model built around target-payload-vehicle-route-biomarker co-design. Within this model, “delivery-adjusted targetability” places spatial accessibility and achievable target engagement alongside clonality and biological dependency, and the target-anatomical compartment pair becomes a practical unit for therapeutic planning. These concepts are not intended as rigid scoring systems; they are decision frameworks that make hidden assumptions explicit.

The field should prioritize trials that measure delivery rather than assume it, distinguish pharmacodynamic failure from target failure, and treat manufacturing and route of administration as integral features of the medicine. The most important advance may ultimately be conceptual, where delivery is not a downstream formulation problem in diffuse glioma. It is part of the mechanism of action, part of target selection, and part of defining clinical precision. Aligning molecular biology with spatial pharmacology offers a more credible path from target discovery to durable patient benefit.

The broader value of this framework is that it changes what should count as sufficient evidence in glioma gene-therapy development. A target should not advance solely because it is molecularly attractive, a vector should not advance because it accumulates in an enhancing tumor, and a trial should not be interpreted only through radiographic response before delivery and target engagement have been demonstrated. By coupling molecular neuro-oncology with spatial pharmacology, delivery engineering, biomarker science, and CMC, the framework provides a common set of falsifiable questions applicable across otherwise dissimilar platforms, including siRNA, genome editors, CAR-T cells, and oncolytic viruses. This approach can identify biologically elegant but spatially implausible concepts earlier, make negative trials more mechanistically informative, and prioritize products that can realistically reach the compartment responsible for recurrence. The next major advance is therefore unlikely to come from one additional target or vector in isolation, but from treating molecular dependency and spatial access as a single therapeutic design problem. In diffuse glioma, clinical translatability is a property of the entire therapeutic system, not of the target, payload, or carrier alone.

## Figures and Tables

**Table 1:** Translational target classes in diffuse glioma and their delivery implications.

Target Class	Representative Examples	Translational Strength	Principal Liability	Preferred Therapeutic Logic
Shared clonal drivers/neoepitopes	IDH1 R132H; H3 K27 alterations; selected fusions	Disease-defining, often stable, enables molecular enrollment	Frequently intracellular; HLA dependence for T-cell recognition	Small-molecule comparator; mutation-directed vaccine; RNA/editing where delivery permits
Tumor-associated surface antigens	IL-13Rα2; EGFRvIII/EGFR; B7-H3; GD2; EphA2	Directly accessible to engineered immune cells	Heterogeneity, antigen loss, normal-tissue expression	Locoregional CAR-T; multi-antigen or engager strategies; logic gating
Transcriptional/epigenetic dependencies	BRD8-p53 network; state-specific regulators	Can expose non-mutational oncogenic dependencies	Plasticity; intracellular delivery requirement	RNA therapeutics; CRISPRi/epigenome editing; tumor-selective expression cassettes
Cell-state/evolutionary liabilities	MES-like, AC-like, OPC-like, NPC-like states; ecDNA-driven programs	Can address resistance phenotypes	Dynamic and non-clonal; state transitions	Adaptive combinations; multi-state coverage; longitudinal biomarker-guided therapy
Microenvironmental dependencies	Myeloid programs; cytokine and immunosuppressive circuits	Can remodel ecosystem and generate bystander effects	Context dependence; systemic immune toxicity	Tumor-homing cellular gene delivery; armed viruses; local cytokine expression
Neural-interface dependencies	Glutamatergic signaling; C1QL1-BAI3-RAC1; BDNF-TrkB	Targets invasion and recurrence niches not captured by core biopsies	Overlap with normal neurobiology; narrow safety window	Compartment-restricted RNA or gene modulation; local postoperative delivery

**Table 2:** Selected clinical and late-translational studies illustrating how target, platform, and route interact.

Platform/Study	Target or Payload	Route/Setting	Stage and Key Translational Lesson
IL-13Rα2 CAR-T [19]	IL-13Rα2	Intratumoral, intraventricular, or dual; recurrent HGG	Phase I, 65 patients. Repeated locoregional dosing is feasible; route, antigen coverage, cell persistence, and CSF pharmacodynamics are integral to product behavior.
Bivalent EGFR/IL-13Rα2 CAR-T [20,94]	EGFR + IL-13Rα2	Intrathecal/CSF-directed; recurrent GBM	Phase I, 18 patients in the updated report. Regression in 8/13 measurable cases, but only 1 confirmed partial response; median PFS 1.9 months. Multi-antigen bioactivity does not ensure durable control.
CARv3-TEAM-E [21]	EGFRvIII CAR + secreted EGFR engager	Intraventricular; recurrent GBM	Early phase I, 3 patients. Illustrates bystander targeting of antigen-heterogeneous disease; efficacy remains unproven.
GD2 CAR-T [18]	GD2	Intravenous and intracranial; H3 K27-altered/M DMG	Phase I experience. Demonstrates CNS activity and highlights inflammation-related neurotoxicity in constrained anatomy.
B7-H3 CAR-T [22,23]	B7-H3	Intraventricular pediatric DMG; intracranial adult recurrent GBM	Phase I adult trial: 15 patients, 72 infusions; disease control in 8/14 measurable cases. Repeat dosing is feasible, but neurotoxicity and uncontrolled survival estimation require careful interpretation.
G47Δ [68]	Replicating HSV oncolysis/immunogenicity	Intratumoral; residual or recurrent GBM	Phase II, small single-arm study; established operational feasibility and supported conditional Japanese approval, but randomized confirmation is lacking.
CAN-3110 [69]	Tumor-selective HSV replication	Intratumoral; recurrent GBM	Phase I. Connects viral biology, antiviral immunity, and intratumoral immune activation with outcome.
DNX-2401 + pembrolizumab [70]	Oncolytic adenovirus + PD-1 blockade	Intratumoral virus + systemic checkpoint inhibitor; recurrent GBM	Phase I/II. Durable activity in a subset, but prespecified response benchmark was not met; biological activity is not equivalent to definitive efficacy.
Toca 511/Toca FC [72]	Retroviral prodrug activation	Resection cavity/recurrent HGG	Randomized phase III negative for OS. A key example of why early signals require spatial PK/PD and controlled validation.
Temferon [31]	Myeloid-restricted IFN-α delivery	Autologous engineered HSPC-derived myeloid cells; newly diagnosed MGMT-unmethylated GBM	Phase I interim analysis, 24 patients. Durable engraftment with low systemic IFN-α; conditioning toxicity and uncertain infiltrative-site exposure remain development constraints.
Personalized DNA vaccine GNOS-PV01 [74]	Patient-specific neoantigens	Systemic vaccination; adjuvant MGMT-unmethylated GBM	Phase I, 9 patients. Multiregional antigen selection and immunogenicity; observed vaccine initiation exceeded the prespecified feasibility window, emphasizing timely product availability.
TGX-007/ADePT [96]	AAV HSV-TK + IL-12	Intratumoral; newly diagnosed or recurrent high-grade glioma	Phase I/II initiated in 2026. Important clinical entry of a dual-payload AAV platform; no efficacy results yet.

## Data Availability

No new data were created or analyzed in this study. Data sharing is not applicable to this article.
